# Data‐driven model optimization for optically pumped magnetometer sensor arrays

**DOI:** 10.1002/hbm.24707

**Published:** 2019-07-11

**Authors:** Leonardo Duque‐Muñoz, Tim M. Tierney, Sofie S. Meyer, Elena Boto, Niall Holmes, Gillian Roberts, James Leggett, J. F. Vargas‐Bonilla, Richard Bowtell, Matthew J. Brookes, Jose D. López, Gareth R. Barnes

**Affiliations:** ^1^ SISTEMIC, Engineering Faculty Universidad de Antioquia UDEA, Calle 70 No 52–51 Medellín Colombia; ^2^ MIRP Research Group, Engineering Faculty Instituto Tecnológico Metropolitano ITM Medellín Colombia; ^3^ Wellcome Centre for Human Neuroimaging UCL Institute of Neurology, University College London London UK; ^4^ Institute of Cognitive Neuroscience, University College London London UK; ^5^ Sir Peter Mansfield Imaging Centre, School of Physics and Astronomy University of Nottingham Nottingham UK

**Keywords:** beamforming, co‐registration, optically pumped magnetometers, source reconstruction

## Abstract

Optically pumped magnetometers (OPMs) have reached sensitivity levels that make them viable portable alternatives to traditional superconducting technology for magnetoencephalography (MEG). OPMs do not require cryogenic cooling and can therefore be placed directly on the scalp surface. Unlike cryogenic systems, based on a well‐characterised fixed arrays essentially linear in applied flux, OPM devices, based on different physical principles, present new modelling challenges. Here, we outline an empirical Bayesian framework that can be used to compare between and optimise sensor arrays. We perturb the sensor geometry (via simulation) and with analytic model comparison methods estimate the true sensor geometry. The width of these perturbation curves allows us to compare different MEG systems. We test this technique using simulated and real data from SQUID and OPM recordings using head‐casts and scanner‐casts. Finally, we show that given knowledge of underlying brain anatomy, it is possible to estimate the true sensor geometry from the OPM data themselves using a model comparison framework. This implies that the requirement for accurate knowledge of the sensor positions and orientations a priori may be relaxed. As this procedure uses the cortical manifold as spatial support there is no co‐registration procedure or reliance on scalp landmarks.

## 1 INTRODUCTION

Optically pumped magnetometers (OPMs) provide a sensitive, flexible and low‐cost alternative to superconducting quantum interference devices (SQUIDs) for measuring magnetoencephalography (MEG) data (Boto et al., 2016). OPMs work by measuring the transmission of laser light through a cell containing a vapour of spin‐polarised alkali atoms, providing a highly sensitive measure of the local magnetic field inside the cell. Unlike the conventional SQUID sensors, OPMs do not require cryogenic cooling and can be placed flexibly on the scalp surface with a minimum separation of ~ 4–7 mm. This potentially offers an increased sensitivity with respect to traditional MEG devices, and makes the MEG system wearable with subjects able to move their head during the measurement (Boto et al., 2018). In this way, OPMs have the potential to form the basis of high signal‐to‐noise‐ratio (SNR) and flexible MEG instrumentation. Unlike SQUID systems, in which intrinsic sensor linearity, cross‐talk and inhomogeneity issues have all been comprehensively addressed; OPM devices, based on different physical principles, present new modelling challenges. Additionally, the flexibility in array geometry introduces technical challenges that must be overcome in order to create a practical, robust and wearable system that can be used in both basic and clinical neuroscience settings. In this article, we outline an empirical Bayesian framework within which to address and build upon these modelling challenges.

A central goal of analysing MEG data is to spatio‐temporally reconstruct the neural sources of the observed signals. However, the spatial specificity of such source reconstruction is highly dependent not only on the data's SNR, but also on having an accurate model of the brain's anatomy and the spatial relationship between the brain and the sensors. The dependence on accurate modelling is even more pronounced with OPMs because increases in SNR (due to the proximity of the sensor to the scalp) also entail increases in sensitivity to modelling errors. In other words, if there is some topographical blurring in the data and large distance between the sensors and sources, a small error in the model of the relationship between the brain and sensors makes little difference, but if the data have very high SNR and the sensors are very close to the brain, small errors in the model lead to distorted estimates of the sources. In (Boto et al., 2016), simulations showed that even small (5%) modelling errors could undermine the four‐fold SNR increase promised by OPM systems.

**Figure 1:**
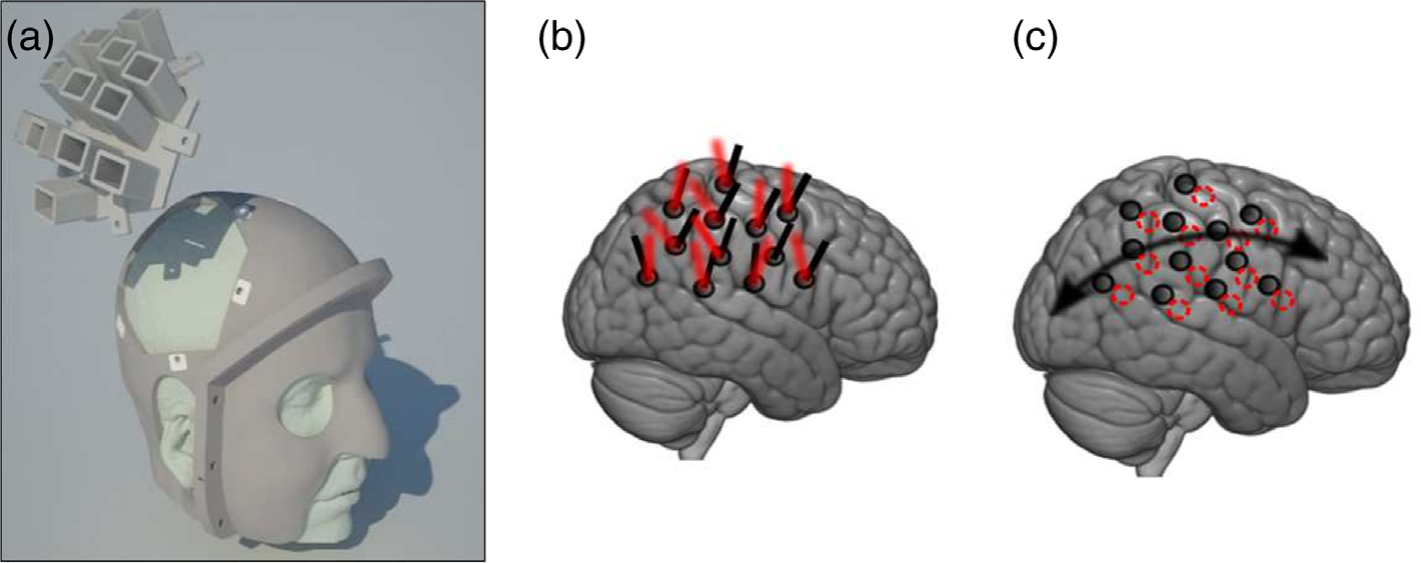
Head‐cast and array perturbations. (a) Digital model of the scanner‐cast. The cast is based on an individual MRI scan and designed to house the OPM sensors around the outer scalp surface. (b) Sensors were independently perturbed from their true orientation (black) by a fixed angle in random direction (red). (c) The rigid sensor array was displaced from its true position (black) to new positions (red) within an arc spanning −20 to 20 mm (and subsequently within a cube of 40 × 40 × 40 mm^3^) [Color figure can be viewed at http://wileyonlinelibrary.com]

The use of OPMs in wearable arrays brings uncertainty to both the absolute and relative sensor locations and orientations. This contrasts with cryogenic systems, where although there is uncertainty on the location of the head (which can be accounted for; López, Penny, Espinosa, & Barnes, 2012), the relative channel locations and orientations are known with a high degree of accuracy. To date, the co‐registration of OPMs with anatomy has been done using classical EEG electrode locations (Sander et al., 2012); using optical scans of the subject's scalp/face matched with known OPM geometry (Zetter, Iivanainen, & Parkkonen, 2019); and recent studies propose the use of a small array of electromagnetic coils of known orientation and location (Pfeiffer et al., 2018). Our local solution has been to minimise the co‐registration problems through the construction of subject‐specific scanner‐casts (Boto et al., 2017), Such casts are three‐dimensional (3D) printed with predefined sensor slots and fit the subject specific head shape (Figure 1a). The scanner‐cast solution is useful for optimising the data quality, as it removes a number of unknowns (see previous works with an MEG head‐cast, Troebinger et al. [2014]), but it is not a necessarily practical solution—as it requires a great deal of investment per‐subject and is both physically cumbersome and intimidating. Thus, in an ideal situation, one would like to be able to use OPMs in flexible wearable arrays like those used for EEG electrodes, but this flexibility in the arrangement of sensors introduces uncertainty about both the absolute and relative sensor locations and orientations.

The motivation of this article is to use the prior knowledge of cortical and volume conductor geometry to estimate the positions and orientations (and error bounds on these estimates) of an array of OPM sensors used to record data. If this is possible, then this procedure reduces the dependence on 3D printed scanner‐casts, suggesting that a more flexible and scalable design can be used to harness the potential of OPMs in a more practical manner. Moreover, it removes reliance on scalp landmarks for co‐registration, and provides an objective test of the quality of our data and forward models (i.e., whether they can be combined to recover the true OPM sensor locations).

To carry out our analysis, we make use of real and simulated data from cryogenic multi‐channel recordings using a head‐cast; single channel OPM measurements using a scanner‐cast (Boto et al., 2017); and simultaneous multi‐channel measurements using the same scanner‐cast. We then perturb our assumptions about the sensor positions and orientations obtaining a forward model for each hypothetical (or true) sensor configuration, and estimate the source distribution on the cortical surface by maximising the model evidence over a range of sensor configurations. The model evidence is approximated by the negative variational Free energy (Friston, Mattout, Trujillo‐Barreto, Ashburner, & Penny, 2007). Each solution gives a single (maximal) Free energy value for each possible sensor configuration. The sensor configuration that can provide the simplest explanation of the magnetic field produced by a current distribution on the individual's cortical surface will have the higher model evidence (Friston et al., 2008). This simplest explanation of the data occurs when the theoretical and empirical sensor geometries are in accord (López et al., 2012). Free energy has similarity to Akaike's and Bayesian Information (Penny, 2012) criteria, which trade accuracy against model complexity. It has been shown to accord with cross‐validation metrics (Bonaiuto et al., 2018), where complex models are penalised by their failure to generalise. It can also be seen as analogous to classical statistical methods (like Chi‐square for dipole fitting [Supek & Aine, 1993]) for determining when an increase in model complexity can be justified.

By using the scanner‐cast (where relative sensor positions and orientations are known, and absolute positions and orientations are known to within ±3 mm and ± 5° respectively) with real measured data, we can directly test this method empirically.

In what follows, we develop a principled method for comparing forward models based on a priori unknown brain activity in which we simply ask how far we could have displaced the sensors before our models (explaining the impact of brain activity on the sensors) become significantly worse.

The article is divided as follows. We first investigate the effect of (independent) sensor orientation error. We show how sensor perturbations away from the true geometry degrade model evidence. This manipulation gives us a metric of how far the modelled sensors in a system can be perturbed before we would notice (for a well‐modelled system with high SNR this should be a small amount). We then use this metric on real data from OPM and SQUID systems where geometry is well known based on scanner‐cast and head‐casts respectively. Finally, we show how it is possible to recover the location of a scanner‐cast sensor array with respect to the cortical surface based purely on the recorded OPM data, and how uncertainty in this estimate can be accounted for in the ensuing source estimate.

## 2 METHODS

We derive a framework to compare measurement systems based on their sensitivity to perturbations in sensor geometry. Based on previous SQUID‐based studies (López et al., 2012; Martínez‐Vargas et al., 2016; Meyer et al., 2017; Stevenson et al., 2014; Troebinger et al., 2014), we would expect that a model of the OPM MEG data with the true geometry will have a higher evidence as approximated by the negative variational Free energy. In this study, as we wish to compare between sensor types (and the data are different precluding any direct comparison of Free energy values), we focus on the sensitivity of the Free energy to perturbations in the geometry. The rationale is that poor models will be less sensitive to this geometrical noise. We first introduce the OPM sensors and the perturbation of the array geometry. Then, we describe how it is possible to score the models with Free energy. Finally, we describe the empirical data collection.

### 2.1 OPM sensors

The QuSpin (http://quspin.com) OPM sensors used here (Shah & Wakai, 2013) have a noise level comparable to SQUIDs (~15 fT/$\sqrt{\mathrm{Hz}}$ above 10 Hz), a bandwidth up to 130 Hz (first order cut‐off), an operational dynamic range of ±1.5 nT, a size of 14 × 21 × 80 mm^*3*^, and can be placed such that the sensitive volume is 6.5 mm from the scalp surface. We modelled the sensitive volume of gas as a single point measurement of field normal to the sensor base. The interested reader on the physical principles of OPMs is directed to other general overviews (Benumof, 1965; Dupont‐Roc, Haroche, & Cohen‐Tannoudji, 1969; Kastler, 1973; Ledbetter, Savukov, Acosta, Budker, & Romalis, 2008).

### 2.2 Scanner‐casts

As a basis for both the simulated and empirical experiments, we used the array geometry as defined in (Boto et al., 2017). Briefly, this relies on 3D printing to construct an individualised helmet containing a sensor array positioned over the subject's sensory motor cortex (Figure 1, for more details, see Boto et al., 2017). As the scanner‐cast was built directly from the subject's MRI, the location and orientation of the cast with respect to the brain anatomy was known to within ±3 mm and ± 5° (conservative estimates based on how far the cast could be manipulated whilst on the subject).

### 2.3 Variations in sensors orientations and locations

To assess whether we could derive the correct sensor geometry based on the OPM data, we perturbed the sensor array in two ways. First, we randomly perturbed the orientation of each sensor independently within the OPM array. For each sensor, the axis of the perturbation (roll, pitch or yaw in *x*, *y* or *z*) was selected randomly and these perturbations were moved in 2.5° steps between −20 and +20° (Figure 1b). Second, we perturbed the sensors either in a one‐dimensional (1D arc around the head from −20 to 20 mm (Figure 1c) or within a 3D volume of 40 × 40 × 40 mm^3^ (see Metropolis section). For each perturbation, we then computed a forward model, estimated the most likely cortical current distribution, and obtained a Free energy value (see next section).

### 2.4 Source reconstruction

We used an empirical Bayesian framework to estimate the underlying cortical current flow given each possible forward model. For a set of MEG signals, the estimation of the current flow *J* involves the computation of an ill‐posed inverse problem, in which the relation between sources and MEG data can be expressed through the general linear model (Dale & Sereno, 1993): *Y* = *LJ* + *ɛ*; where $Y\in R^{\left(N_{c}\times N_{t}\;\right)}$ are the measured MEG data with *N*_*c*_ channels and *N*_*t*_ time samples affected by zero mean Gaussian noise $ɛ=N\left(0,Q_{ɛ}\right),ɛ\in R^{\left(N_{c}\times N_{t}\;\right)}$, and the noise covariance $Q_{ɛ}\in R^{\left(N_{c}\times N_{t}\right)}$. $J\in R^{\left(N_{d}\times N_{t}\;\right)}$ is the current flow due to *N*_*d*_ current dipoles distributed across the cortical surface, with prior Gaussian assumptions *J* = *N*(0, *Q*). $Q\in R^{\left(N_{d}\times N_{d}\right)}$ is the source level covariance matrix. The gain matrix $L\in R^{\left(N_{c}\times N_{d}\right)}$ (commonly known as the lead‐field matrix) contains a model of the magnetic fields that would be measured at each sensor in response to a current source of unit amplitude within the cortical surface.

We adapted the framework presented in López et al. (2012) to explore across the perturbed arrays. Briefly, with each perturbed array a new gain matrix *L*_*a*_ (forward model) was computed using the SPM12 software package (http://www.fil.ion.ucl.ac.uk/spm/) using predominantly the single shell forward model (Nolte, 2003), although we also made use of the single sphere model (Hämäläinen & Sarvas, 1987). For uninformative priors, the Maximum‐likelihood solution to the inverse problem reduces to:

(2) $$\hat{J}=QL_{a}^{T}{\left(Q_{ɛ}+L_{a}QL_{a}^{T}\right)}^{-1}Y$$

where each lead field *L*_*a*_ can be computed based on the sensor and volume conductor geometry. This means that the sensor‐ and source‐ level covariance priors are critical to estimate the source amplitudes *J*. As such, we assume the sensor noise to be homogenous across sensors and independent (see Section 4), i.e., *Q*_*ɛ*_ = *λ*_1_*I*, with *λ*_1_ a regularisation parameter. For the source level prior covariance, we use a weighted covariance estimate based on an empirical Beamformer prior (based on the sensor covariance). This Empirical Bayes Beamformer (EBB; Belardinelli, Ortiz, Barnes, Noppeney, & Preissl, 2012) makes a direct estimate of the source level covariance based on the assumption that there are no zero‐lag correlated sources. Empirically, we used all the available MEG channels and the 0–300 ms (2–80 Hz) post‐stimulus period in each data set for the inversions: the 13 OPMs and the 275 sensors in the SQUID data.

### 2.5 Free energy as objective function for model selection

In this work, we use the Free energy to score competing source reconstructions based on different sensor locations and orientations (modelled through different *L*_*a*_ models). That is, reconstructions of the same data but with different sensor configurations, each providing an associated Free energy that can be compared across geometries (Henson, Mattout, Phillips, & Friston, 2009). For a model *L*_*a*_ associated with a given sensor location and orientation, Free energy *F*_*a*_ can be expressed as a trade‐off between accuracy and complexity:

(5) $$F_{a}=\text{Accuracy}\left(a\right)-\text{Complexity}\left(a\right)$$

The accuracy is expressed as:

(6) $$\text{Accuracy}\left(a\right)=\frac{N_{c}}{2}\text{trace}\left(C_{Y}C_{a}^{-1}\right)-\frac{N_{c}}{2}\log\left|C_{a}\right|-\frac{N_{c}N_{t}}{2}\log\left(2\pi \right)$$

where $C_{Y}=\frac{1}{N_{c}}YY^{T}$ the data‐based sample covariance matrix, *N*_*t*_ is the number of samples, and ∣·∣ is the matrix determinant operator. When searching for the optimal geometry, the MEG data do not change; so, the accuracy of the model *a* mainly depends on the model‐based sample covariance matrix computed as $C_{a}=Q_{ɛ}+L_{a}QL_{a}^{T}N_{t}$.

In the EBB algorithm, the complexity term depends on the hyperparameters *λ* that provide a trade‐off between the sensor noise $Q_{ɛ}=\lambda_{1}I_{N_{c}}$, and the Beamforming prior *Q*_*a*_ = *λ*_2_Γ, with Γ being the beamforming prior:

(7) $$\text{Complexity}\left(a\right)=\frac{1}{2}{\left(\hat{\lambda_{a}}-\nu \right)}^{T}\Pi \left(\hat{\lambda_{a}}-\nu \right)+\frac{1}{2}\log\left|\sum_{\lambda_{a}}\Pi \right|$$

The prior and posterior distributions of the hyperparameters are considered Gaussian: *q*(*λ*_*a*_) = *N*(*λ*; *ν*, Π^−1^) and $p\left(\lambda_{a}\right)=N\left({\hat{\lambda }}_{a},\sum_{\lambda_{a}}\right)$, respectively (where ${\hat{\lambda }}_{a}$ and ∑_*λ*_ are the posterior mean and covariance of the hyperparameters for a model *a*).

We used the standard SPM implementation of this algorithm with non‐informative mean and precision (*ν* and Π) by casting these terms as identity matrices scaled close to zero mean and with very low precision respectively, to provide a non‐informative prior.

### 2.6 Metropolis search and Bayesian model averaging

Free energy provides a metric to judge between different sensor geometries but a systematic search over the possible space of geometries would be extremely time‐consuming. We, therefore, chose the Metropolis search (Gelman, Carlin, Stern, & Rubin, 2000) to deal with the possibly high non‐linear non‐convex search space. The metropolis search is an optimisation strategy that follows a Markov chain with variable step given a probability distribution centred on the last step. Following the adapted Metropolis search strategy proposed in López et al. (2012), the parameters of the Markov chain are updated to follow increasing Free energy values, but decreases are also allowed to avoid local maxima. The detailed version used here is presented in Appendix METROPOLIS SEARCH.

Although the metropolis search provides a single global maximum, the sensor geometry at this point may have very similar model evidence to geometries nearby. The use of Bayesian model averaging (BMA) allows us to combine the estimates from all geometries in a principled way. This adds robustness to the solution (as it is no longer based on a single geometry) and allows us to directly compute confidence bounds. With this framework, we are thus able to make inferences about the array location with the Metropolis search, and inferences about functionality by using BMA across a range of array locations. As such,

(8) $$p\left(J|Y\right)\approx \sum_{k}p\left(J|Y,h_{k}\right)p\left(h_{k}|Y\right)$$

where *p*(*J*| *Y*, *h*_*k*_) is the distribution of the sources obtained with model *h*_*k*_. This is evaluated using

(9) $$p\left(J|Y\right)\approx \sum_{s}p\left(J|Y,h_{s}\right)$$

where *h*_*s*_ are the posterior samples produced with Metropolis search algorithm. The BMA implementation used here is detailed in Appendix BAYESIAN MODEL AVERAGING.

### 2.7 Task

We used empirical data from a somatosensory evoked response paradigm, which involved electrical stimulation of the subject's left median nerve. There are three data sets collected from the same subject and using the same paradigm used in this article:(a) data collected with a SQUID system using a head‐cast, (b) using multiple repeats of the same experiment with a single OPM channel at different locations, and (c) using an array of 13 OPM channels operating simultaneously.

Briefly, we performed a median nerve electrical stimulation by applying a series of 500 μs duration current pulses to two gold electrodes placed on the subject's left wrist. The current was applied using a Digitimer DS7A constant current stimulator, and the amplitude was increased until a visible movement of the thumb was observed upon stimulation. For the single channel OPM data, the ISI was 1.9 s. For the multi‐channel OPM data and the SQUID data, the ISI was 0.5 s. In all cases, we used data sets based on the average of 100 trials. The OPM data sets were based on left median nerve stimulation whereas in the SQUID‐head cast data the right median nerve was stimulated.

All recordings were carried out inside a magnetic shielded room comprising two layers of mu‐metal and one of aluminium. The single OPM measurements made with a sequential sampling of scanner‐cast slots using a single OPM channel are explained in Boto et al. (2017). The multi‐channel recording with the 13 sensors located in the same slots of the scanner‐cast was performed with the subject sitting upright. The same scanner‐cast of Figure 1 was used for both sets of OPM recordings. The single channel OPM data were acquired simultaneously with SQUID data (from a 275 channel CTF instrument), and the magnetometer reference channels within SQUID system (remote from the subject) and the time‐derivatives of these channels were used as an environmental noise reference set and regressed out of the OPM data on a trial by trial basis (as described in Boto et al., 2017). For the multi‐channel OPM recording, we used a similar procedure but used four OPM reference sensors displaced from the main array as reference channels. In the multi‐channel recordings, in which the head was free to move, we used the set of bi‐planar nulling coils positioned either side of the subject in order to minimise the ambient field around the subject's head (Holmes et al., 2018). Although we did not explicitly measure head movement, we estimated it to be 2 cm based on the field changes (0.1 nT, that could not be explained by the fixed reference set) and the known field gradients (Holmes et al., 2018) within the room.

SQUID recordings were performed using the 275 channel system in third gradient configuration (i.e., with factory‐set linear weighting from the noise reference array).

### 2.8 Simulated data

We used the SPM12 software package to simulate single trial OPM‐MEG data sets and to perturb the sensor geometries within the empirical OPM and SQUID recordings. The OPM simulated trials had a 1 s duration consisted of 13 channels (*N*_*c*_ = 13). We simulated a single 10 Hz sinusoidal source located in the somatosensory cortex (at 46, −25, 60 mm in MNI space) with a dipole moment of 10 nAm. We then added the Gaussian white background noise of standard deviation 100 fT RMS to the sensor level data.

## 3 RESULTS

**Figure 2:**
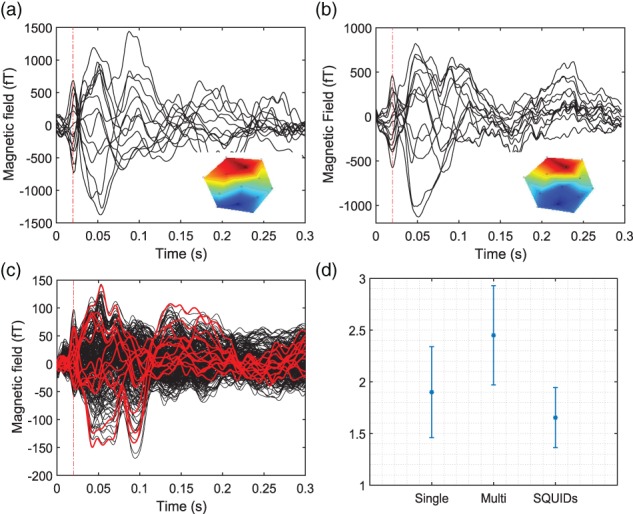
Empirical data. Sensor‐level evoked responses for median nerve stimulation recorded with OPMs and SQUIDs. The N20m evoked response is highlighted (red dotted). (a) Single channel OPM used to sequentially record responses at the 13 different locations across the scanner‐cast, treated as a simultaneous measurement. (b) Multi‐channel OPM data with 13 channels in the same scanner‐cast. The field maps computed for the single and multichannel arrays are embedded in (a) and (b). (c) Conventional SQUID recordings using head cast with the 13 most highly correlated channels to OPM channels highlighted in red. Note the scale changes between sensor types. (d) SNR calculated for the three experiments (using just the 13 SQUID channels). Where SNR = max_*i*_ $\left(\left[\sum_{\mathrm{pre}}Y_{i}^{2}\right]/\left[\sum_{\text{post}}Y_{i}^{2}\right]\right).$ OPMs, optically pumped magnetometers; SQUIDs, superconducting quantum interference devices [Color figure can be viewed at http://wileyonlinelibrary.com]

Figure 2 shows the averaged time courses from single channel OPM data (Figure 2a), multi‐channel data (Figure 2b), and SQUID data (Figure 2c); time zero corresponds to the median nerve stimulation impulse. The expected N20m evoked response is visible with the three experiments (red‐dotted line). Note the scale changes in the axes with the OPM signals being 5–10 times larger in magnitude. As expected, the magnetic fields measured with the OPMs have a stronger response due to their proximity to the scalp; despite this signal magnitude advantage, however, the relative SNRs (at sensor level) are comparable across all three experiments (Figure 2d), this is partly due to the greater intrinsic noise of the OPMs (a factor of 2) and possibly also because much of the variability in the signal is of neural origin.

### 3.1 Analysing the effect of the head model

**Figure 3:**
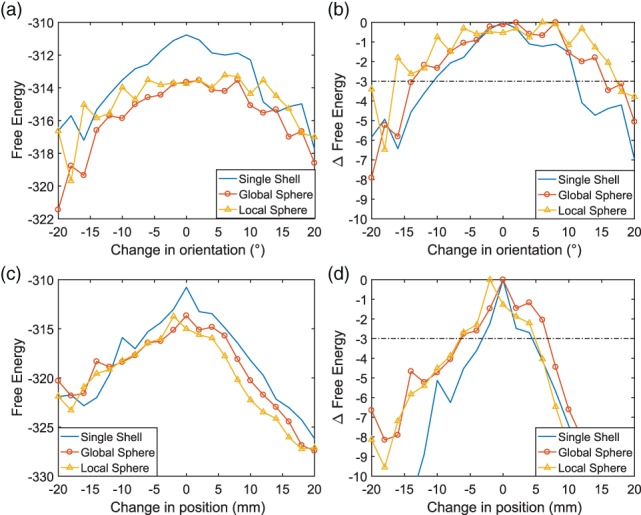
Estimating sensor optimal sensor geometry using different volume conductor models with global sphere (red circles), local sphere (yellow triangles), single shell (blue solid), based on single channel optically pumped magnetometers (OPM) data. Top panels (a,b) show addition or orientation error to individual sensors; lower panels (c,d) show addition of position error to the sensor array. The Free Energy peaks at zero (corresponding to the true sensor geometry) for the single shell and global sphere. Left panels (a,c) show absolute free energy differences between models. The most likely geometry is that of the scanner‐cast (at 0) and the most likely volume conductor, given the data, is the single shell. Right panels (b,d) show the same data normalized to maximal Free energy (the format used later in article when comparing models fit to different datasets)—in this case the width of the peak (or the amount of geometrical noise that could be added to the sensor before a significant degradation in the model) is used to quantify performance. In this case note that the poorer models (the sphere models) are less sensitive to added geometrical noise [Color figure can be viewed at http://wileyonlinelibrary.com]

**Figure 4:**
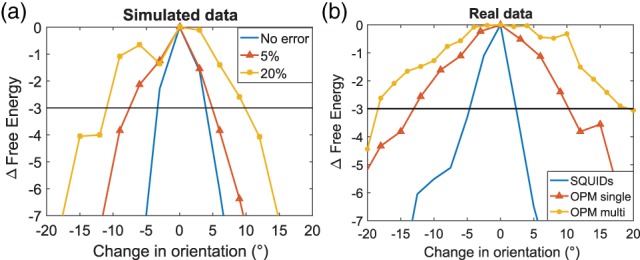
Orientation perturbation curves. Sensitivity of model fit (Free energy metric) to errors in sensor orientation: for perfect sensors (blue solid), sensors with gain errors of 5% (orange triangles), and gain errors of 20% (yellow circles). Adding gain error to the data results in broadening of posterior estimate on sensor orientation. Solid black line (*F* = −3) is the point at which the models become 20 times less likely than the best model. (b) Sensitivity of model fit to orientation errors added to real sensor recordings: for SQUID data (blue solid); single channel OPM data (orange triangles) and multi‐channel OPM data (yellow circles) [Color figure can be viewed at http://wileyonlinelibrary.com]

In order to demonstrate the approach, we first used different volume conductor models to explain the single channel OPM data as geometrical distortion was added. Here the data remain constant allowing us to directly compare models using Free energy; in the subsequent sections, we will be examining changes of relative free energy (with different sensors and data) and so we also show these here for comparison. Figure 4 shows how Free energy varies as a function of added geometrical noise under different volume conductors for the single channel OPM data. We added orientation (a,b) and position c,d) error to the array with a single spheres (Hämäläinen & Sarvas, 1987) fit to the global inner‐skull surface; or fit to the local inner‐skull curvature proximal to the right somatosensory cortex; and single shell (Nolte, 2003) models. Left panels show absolute free energy. Right panels show relative (normalised to maximum) free energy. Figure 3a shows that the models with peak Free energy (or most likely models given the data) have zero orientation error (although the algorithm has no knowledge of true orientation) and that the most likely head model (with the highest Free energy) is the single‐shell. These results are in accordance with Stenros, Hunold, and Haueisen (2014), in that the single shell model outperforms the spherical ones. That said, we were surprised to see such a clear distinction with a relatively small number (13) of sensors. The same data is presented in Figure 3b in terms of relative Free energy in order to provide an analogue to the sections which follow (in which absolute Free energy values cannot be compared). In this case we look at how much the sensor geometry could be degraded before the evidence for the data degrades significantly. The better model (in this case single shell) degrades more rapidly in the presence of geometrical error. Figure 3c,d show the same (absolute and relative) effects for displacements of the sensor array. The single shell model is consistently the most likely, but it is notable that even the simpler volume conductor models could be used to estimate array geometry; although with marginally less accuracy (e.g., note the local sphere model peaks 2 mm offset from zero in Figure 3d).

### 3.2 Adding sensor orientation error

In the first instance, we wanted to examine the sensitivity of our models to sensor orientation error and gain error. The logic being that sensitivity to error in the geometry is a prerequisite for any scheme seeking to optimise geometry. We also considered gain error to account for other un‐modelled sensor imperfections due to calibration or cross‐talk issues. Individual sensor orientations were perturbed by orientation errors between −20 and 20° in a random direction around their true orientation in steps of 2.5°. A total of 30 models were obtained for each orientation error (i.e., each model has all channels perturbed in a different random direction about their true axis by this amount). Additionally to orientation error, we perturbed the models with gain errors of 5 and 20% (Figure 4a).

Figure 4a shows the change in Free energy as a function of channel orientation error for simulated data. The solid line shows simulations with an idealised OPM sensor array. Note that the Free energy peaks at zero error where the measured data can be most simply reconciled with the single generating source. For Free energy values (on a log scale), −3 corresponds to models that are 20 times less likely. In the ideal sensor case, we are, therefore, able to reject sensor geometries with more than ±4° of intrinsic error as unlikely. Also shown are the effects of additional random gain error (5 and 20%; red triangles and yellow circles, respectively) which serve to blunt the orientation perturbation curves. Adding 20% gain error to the sensors means that it is now only possible to confidently reject sensor geometries with greater than ±12° orientation error, although the most likely sensor geometry remains the true geometry. Figure 4b shows the same orientation perturbation curves but based around real measured data from the three MEG systems. All three data sets are also sensitive to perturbations of the geometry of the measurement sensors and suggest that the most likely orientation is the true one. The model used to describe the SQUID data is sensitive to orientation error of less than ±6°, the models used to describe the concatenated single channel OPM data being sensitive to orientation error of ±15° and the model used to describe the multi‐channel OPM data is sensitive to orientation error of ±20°. We speculate (see also Section 4) that the difference between the single and multi‐channel system OPM curves is that the concatenated single channel system is effectively a more homogenous system than the multi‐channel system. The multi‐channel system will suffer from sensor cross talk and other factors (such as calibration, different intrinsic noise levels, etc.) of between‐sensor variability. However, the difference between the SQUID and OPM curves ran counter to our expectation, which was that the OPM models would have the higher sensitivity to perturbation because of the marginally higher SNR (Boto et al., 2016; Hillebrand & Barnes, 2003).

### 3.3 Movement of sensor array

**Figure 5:**
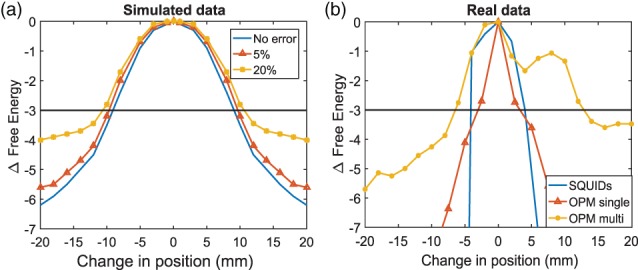
Effect of sensor array displacement on goodness‐of‐fit, simulated and real data. (a) Simulated data. Sensitivity of Free energy to errors in sensor array position: for perfect sensors (blue solid), sensors with gain errors of 5% (orange triangles), and gain errors of 20% (yellow circles). Adding gain error to the data results in subtle broadening of posterior estimate of the sensor array position. (b) Sensitivity of Free energy to array position (ground truth based upon head and scanner‐casts estimates) added to real sensor recordings: for SQUID data (blue solid); single channel OPM data (orange triangles) and multi‐channel OPM data (yellow circles) [Color figure can be viewed at http://wileyonlinelibrary.com]

The other scenario we considered was a rigid array of sensors of known relative geometry attached to the scalp. In this case, the goal is to estimate the array location (as fixed whole) relative to the subject's anatomy. This could be locating a small array of OPMs strapped to the scalp surface or estimating the position and orientation of a generic helmet (e.g., bicycle helmet) containing the sensors. We begin by demonstrating the change in Free energy, as the sensor array is moved in an arc about its true position. Figure 5a shows the effect of this movement on simulated OPM data. Again, the most likely array location is the true location, and the 95% confidence bounds on this location are ±10 mm. As gain error is increased, these error bounds become larger.

Figure 5b shows the same (software) displacement of the sensor array used to collect real OPM and SQUID data (i.e., error was added to the sensor array locations from a real data recording, and a search across a range of array locations was performed with the algorithm being agnostic to the true array location). Again, we were encouraged to find that all models to explain these real data exhibit maximal model evidence at the true array location, although this location is unknown to the algorithm. Here the single channel OPM data and SQUID systems are broadly in accord. For these data sets, models perturbed by more than 5 mm are significantly less likely. In contrast, the OPM multi‐channel recordings have a much broader tuning curve and are relatively insensitive to models perturbed by as much as 10–15 mm.

### 3.4 Model optimization

The practical problem is now to demonstrate how it is possible to locate a rigid sensor array with only approximate positional information based only on the field measurements, a volume conductor model and the cortical geometry. We did this using the data from the single‐channel OPM array in two ways. First, using a simple 1D search passing over the known location of the sensor array. Second, by assuming an initial uniform uncertainty over a 64 (4 × 4 × 4) cm^3^ volume a priori knowledge of sensor array location in any dimension.

### 3.5 Optimisation in one dimension

**Figure 6:**
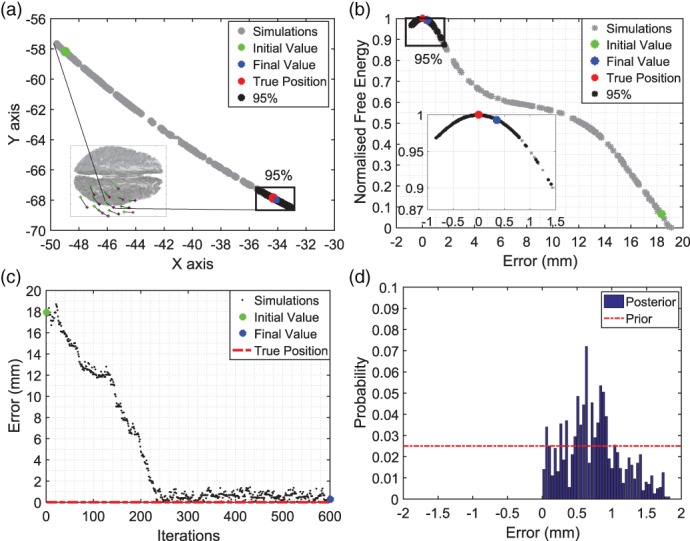
Optimisation in one dimension. (a) Movement of the array through each iteration of the Metropolis search, the array moves through an arc in a 2D plane; the initial value is in 18 mm of error (green point), and evolves through each iteration (grey/black points) until the final estimate (blue point). (b) Evolution of the Free energy through each iteration. A first model is computed with the array centred at the initial value (green point), then the inverse problem is solved and a Free energy value is computed. The position of the array is updated through each iteration of the metropolis search until convergence (black points). The blue point represents the final position of the array while the red point represents the true position (as estimated from the scanner‐cast). (c) Evolution of the distance error from the scanner‐cast location, this error is unknown to the algorithm. (d) Prior and posterior distributions of the array location (based on MEG data and uniform priors); zero represents the estimated array position based on the scanner‐cast [Color figure can be viewed at http://wileyonlinelibrary.com]

We used the Metropolis search algorithm detailed in the Appendix. Four chains were simulated with single axis movement in which the algorithm had no information of the true array position respect to the brain, that is, flat priors on location within *σ* = 40 mm. The Metropolis search was performed with 600 iterations per chain in four chains. Figure 6a shows the change in the position of the array as it moved through an arc of 18 mm. The initial value is represented with a green point. Through each iteration of the Metropolis search (black points), the position changes (via model comparison Figure 6b) until convergence (blue point). The error drops with each iteration (Figure 6c) and after 250 iterations, the algorithm oscillates near to the true position. Figure 6d shows the prior and posterior estimate of the array location. The figure shows that the model estimate of the array position was ~0.6 mm from our estimate of location based on the scanner‐cast. The uncertainty (95%) on this geometry estimate is also less than ±1 mm (panel d).

### 3.6 Optimisation in three dimensions

**Figure 7:**
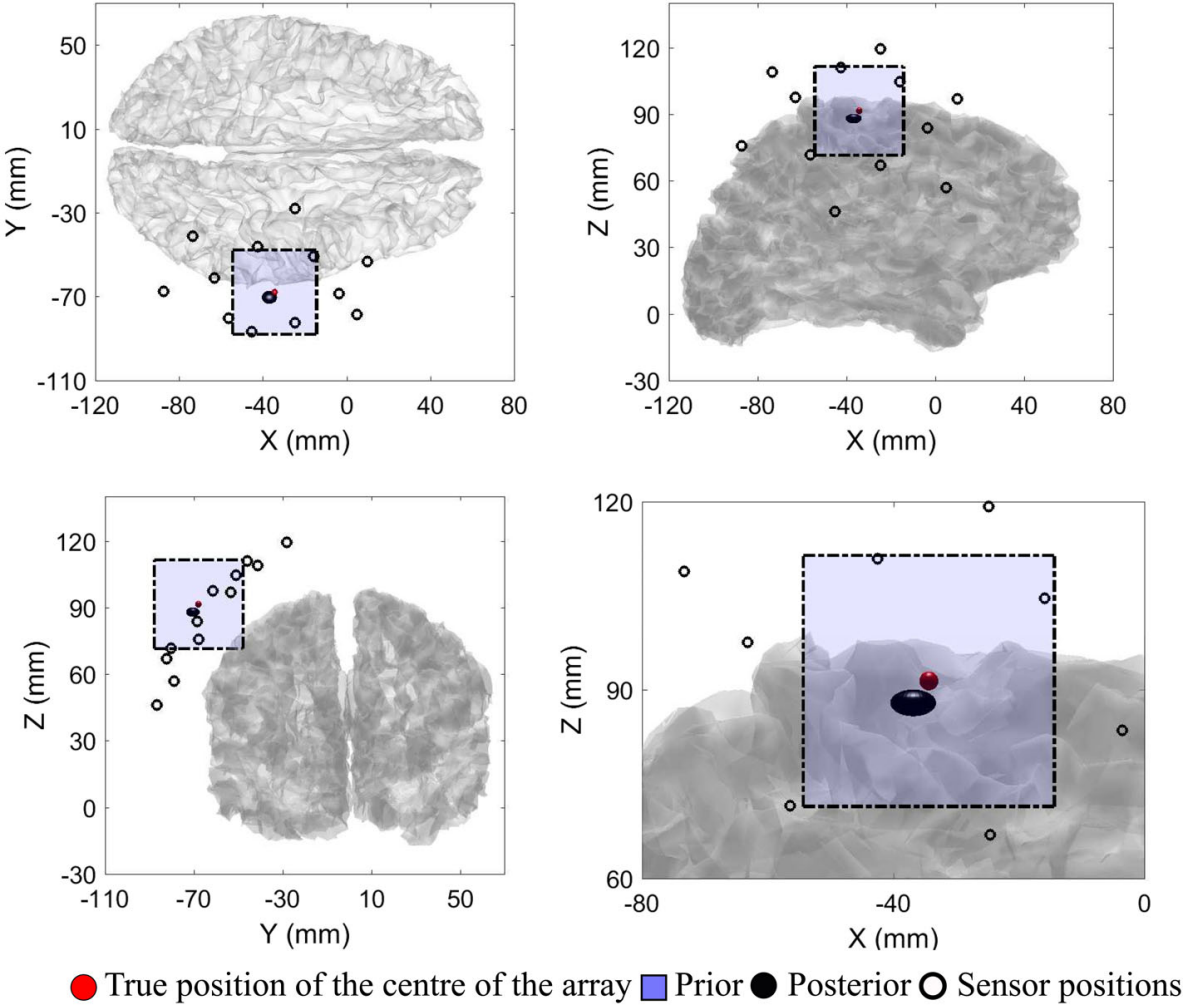
Optimisation in three dimensions (sensor space). The dotted cube shows the original 4 x 4 x 4 cm^3^ uncertainty on array location. The 95% confidence ellipsoid (black) shows the posterior location of the central sensor (and hence the whole rigid array). The location of the central sensor based on the scanner‐cast information is show as a red dot. Lower right panel is a magnified sagittal view [Color figure can be viewed at http://wileyonlinelibrary.com]

Although the optimization in 1D provides a clear illustration of the Metropolis process, it is not practically useful since positional uncertainty will rarely be constrained to lie in one dimension. To show how this method can be generalised to higher dimensional spaces we used the same Metropolis procedure but based on the assumption that sensor location was only known to within an approximate 3D volume of 4 × 4 × 4 cm^3^. Figure 7 shows the prior cubic volume for the central sensor in the array (blue); alongside the posterior confidence interval (black ellipsoid) and the scanner‐cast estimate of this sensor location (red). The Metropolis search and BMA estimated the posterior mean array position to be 4 mm displaced from that we expected from the scanner cast. The posterior confidence volume on this location was 0.1019 cm^3^, that is, a 600‐fold reduction on the prior volume.

**Figure 8:**
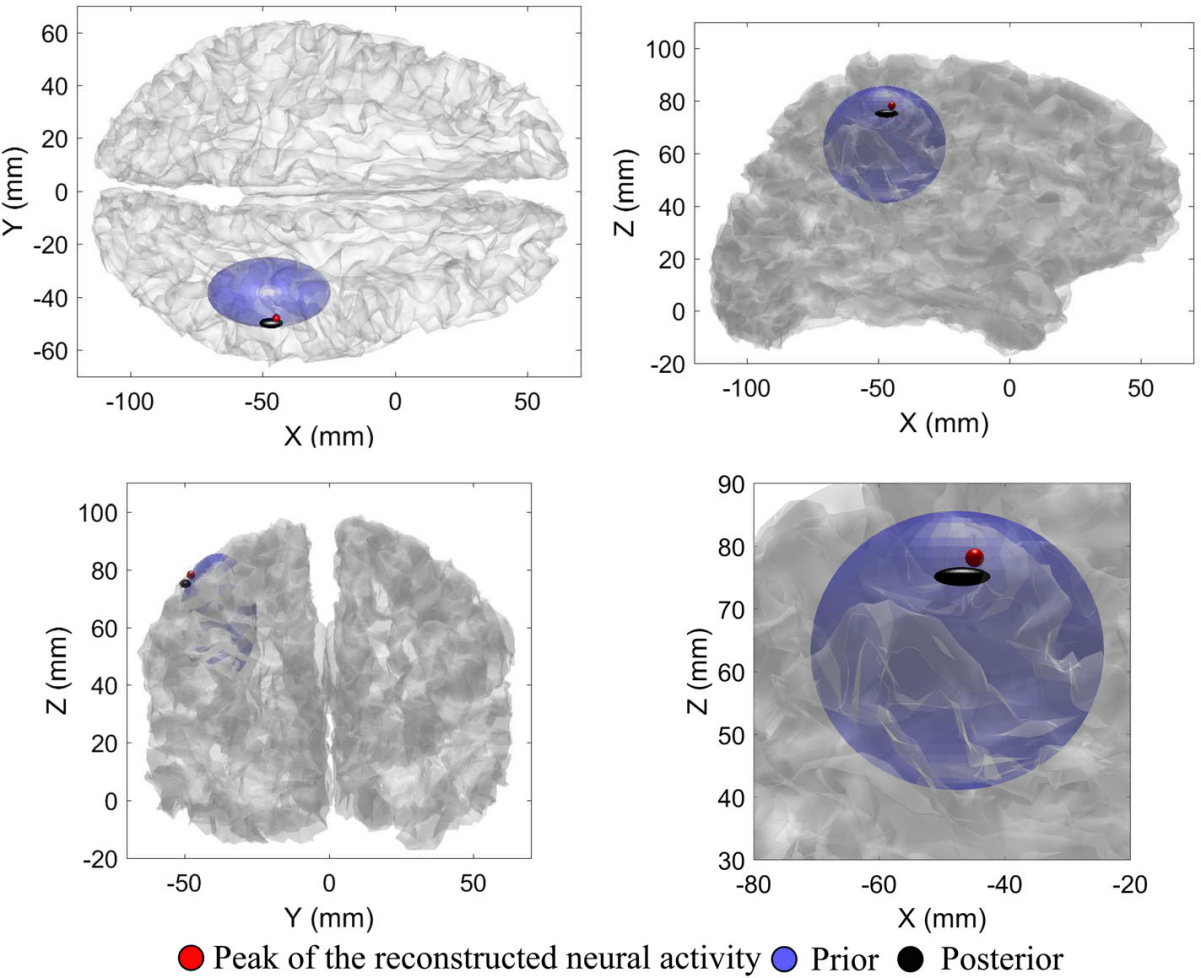
Optimisation in three dimensions (source space). Source estimates with confidence volumes shown in three orthogonal views. The red sphere represents the peak of the reconstructed neural activity when reconstructed with sensors at the scanner‐cast locations. The initial sensor uncertainty gives rise to a prior distribution on the peak of the electrical activity (blue ellipsoid; based on reconstructions over 30 sensor locations distributed randomly across the prior volume). The black ellipsoid is the posterior estimate of electrical activity after BMA. The estimated source location when the sensor array location is unknown is 5 mm from the peak source location as estimated using the scanner‐cast information [Color figure can be viewed at http://wileyonlinelibrary.com]

It is also possible to view the consequence of the refinement of sensor position at the source level. Estimating the source level activity based on our prior knowledge of sensor position (4 × 4 × 4 cm^3^), gives a distribution of (of peak locations) than can be described by the 95% by the confidence ellipsoid (blue) in Figure 8. With the BMA step, we are able to pool estimates from across a range of optimisation steps and weight them by their model evidence. This gives a degree of robustness to the process and importantly provides us useful posterior estimates of the head location and an estimation of current distribution with a confidence interval. Also shown in Figure 8 is the posterior confidence volume on the peak source location after the BMA over sensor geometries (black). The model optimization reduces the confidence volume on peak location from 34.90 cm^3^ to 0.05654 cm^3^. The centre of the optimised confidence volume is 5 mm from the source estimate when using the scanner‐cast location as ground truth (red dot).

## 4 DISCUSSION

OPM sensors are rapidly decreasing in size (Alem, Benison, Barth, Kitching, & Knappe, 2014); and lightweight, multi‐channel wearable arrays will soon become of clinical use (Boto et al., 2018; Tierney et al., 2018). To date, we have maximised the utility of OP‐MEG data for neural source reconstruction by minimising sensor position uncertainty a priori using scanner casts (see Boto et al., 2017). Here we have developed a framework, which will allow us to compare between different OPM device and array models. The fundamental observation is that better models will degrade more rapidly as simple geometrical errors are introduced. We show how we can also exploit this framework to recover the true sensor geometry based on the recorded MEG data and a subject specific head model. This approach could reduce the dependence on rigid, time‐consuming and somewhat intimidating 3D printed scanner casts, and potentially gives way to a more EEG‐like system that is flexible, comfortable, and easier to use.

Here, we again showed how model evidence is a useful metric to judge not only the quality of the source reconstruction (Friston et al., 2008; López, Litvak, Espinosa, Friston, & Barnes, 2014) but also the quality of the forward model (Henson et al., 2009; López, Valencia, Flandin, Penny, & Barnes, 2017). Model evidence is however data‐dependent and cannot be compared across data sets or MEG systems. Here, we introduced the idea of quantifying how sensitive a given system is to geometrical perturbation. We demonstrated how the width of these perturbation curves could be used to compare different MEG systems or MEG system architectures. Other strategies have been proposed, Lau, Yam, & Burneo, 2008 compared optimization strategies for sensor spacing using the condition number of the gain matrix as a cost function; whereas Eichardt et al., 2012 proposed a modified condition number to optimise the source grid for imaging magnetic nano‐particles. Other authors have focused on metrics of dipole localization error (Vrba & Robinson, 2002), or point spread functions (Boto et al., 2016; Livanainen, Stenroos, & Parkokonen, 2017).

An important use of this method will be to refine the models of the OPM sensors themselves. For example, the single channel OPM measurements were considerably more sensitive to orientation error (Figure 4b) and position (Figure 5b) than their multi‐channel counterparts. We would expect that as our models of the multi‐channel array improve (by accounting for cross talk, gain inconsistencies, etc.) we will observe a tightening of these perturbation curves. At the moment, we can think of several possible reasons why the models of multi‐channel data are suboptimal (Figure 4b). First, the multi‐channel system will suffer from cross talk which we estimate to be around 3% (Boto et al., 2018). Second, we made the assumption that the sensor noise covariance matrix *Q*_*ɛ*_ is a scaled identity matrix (i.e., same noise in all sensor). For the single (repeated) sensor measurements, it is reasonable to assume so, but we estimate the white noise floor (RMS fT/√Hz) across the multi‐channel array varied by around 16%.Third, the multi‐channel OPM measurements took place with the subject sitting and the head unconstrained. Although we used a bi‐planar coil set to minimise fields and field gradients around the head, there was an average of 0.1 nT change in field across all sensors during the measurement (which could not be explained by the static reference sensors) this would give rise to changes in the calibration (applied field vs. output voltage) of the order of 0.1%. Here we used the Empirically Bayesian Beamformer (Belardinelli et al., 2012) for source reconstruction. This formulation uses a beamformer estimate of source power as the prior for the subsequent empirical Bayes optimization. This beamformer estimate (of the prior) depends on the inversion of a data‐covariance matrix, which we know can be optimised through regularisation (Engemann & Gramfort, 2014; Woolrich, Hunt, Groves, & Barnes, 2011). In this case, we used a fixed regularisation of zero which could give rise to sub‐optimal source covariance priors (the condition numbers for the single and multi‐channel OPM systems were 40.31 and 4.62 × 10^4^ respectively).

Although all of the data were collected from the same individual wearing either scanner or head cast, there were however differences in the recording paradigms. First, the SQUID data were collected based on right rather than left median nerve stimulation. Secondly, the ISI for the multi‐channel OPM and SQUID measurements was 0.5 s, in contrast to 1.9 s for the single channel OPM data which we know will influence the evoked response components profile (Wikström et al., 1996). We, therefore, cannot rule out that there is some disparity in how well the data are modelled at the source level, which could in turn change the steepness of the geometrical tuning curves. We also tested the possibility that the SQUID tuning curves to position and orientation might benefit from the 5 cm baseline axial gradiometer configuration, but found negligible theoretical difference.

The problem of uncertain sensor placement is not specific to OPM MEG. Dalal, Rampp, Willomitzer, and Ettl (2014) have shown that inaccuracies of EEG electrode coordinates form an error term in the forward model and ultimately in the source reconstruction performance. This error arises from the combination of both intrinsic measurement noise of the digitization device and manual co‐registration error when selecting fiducials on anatomical MRI volumes. OPMs pose additional challenges over EEG in that neither orientation nor position will be known in a more flexible set‐up. These problems will be yet more acute for the OPMs because the sensitivity to modelling errors is highly dependent on SNR (Boto et al., 2016; Dalal et al., 2014; Hillebrand & Barnes, 2003).

In this study, we have approximated the OPM as a point measurement system. In reality, the volume of the gas exposed to the laser light has maximal dimension of 3 mm. This distance is relatively large given that the OPM sensors may now sit <20 mm from the brain. The addition of appropriate integration points within this volume would be a useful avenue for further study.

In addition to more precise sensor characterisation, the increased spatial sampling and sensitivity offered by OPMs will certainly demand more complex head models. Boto et al. (2016) already showed that small gain errors can forsake all potential advantages of OPMs over SQUIDs. Here we have shown that the Nolte single shell model consistently performed better than single and multi‐sphere counterparts. As the technology matures, with larger sensor arrays and longer recording times, we will expect to move from realistically shaped three shell models Stenros et al. (2014) to the inclusion of more complex models with cerebrospinal fluid, skull spongiosa and conductivity anisotropy (Vorwerk et al., 2014). We should note that the method is not simply bounded by the forward model; we know from previous work that the inversion assumptions will also constrain the accuracy of the solution (Stevenson et al., 2014; López et al., 2017; Little et al. 2018).

We have demonstrated how the spatial parameters (position and orientation) of a sensor array can be physically characterised based on magnetic fields derived from the human brain. In addition to removing the dependence on a scanner‐cast, we can also dispense with traditional co‐registration procedures and the associated subjective identification of scalp landmarks. The co‐registration here is performed with respect to inner skull anatomy (cortex and inner skull boundary) and unlike typical co‐registration procedures, the geometrical uncertainty is directly factored into the source estimate giving realistic confidence bounds (see also López et al., 2012). For example, the experimenter needs only to specify that the array is approximately above the right ear (with a 64 cm^3^ volume) and the algorithm is able to reduce this uncertainty by 600‐fold to 0.1019 cm^3^. One issue, which remains to be tested is how well the estimate of array position will generalise across the scalp surface. It may well be that the method is challenged in regions where the forward model is poorly specified (e.g., frontal lobes) or where the generative model is complicated (e.g., the cerebellum).

Here we used only a three parameter optimization of a fixed array but the algorithm directly generalises to optimization over much larger parameter spaces (for example when only the topology of the array is known). The main consideration being the additional amount of data required. Importantly, as OPM devices are becoming wearable, we can expect subjects to tolerate the scanning environment for considerably longer periods, we will likely have far more data available with which to perform such optimizations. This would mean that the physical characterisation of the sensor array and optimization of forward models could be performed on data orthogonal to that under‐scrutiny. For example, using stationary parcellations of resting‐state data (Martínez‐Vargas et al., 2016). Additionally, we have measurements of magnetic fields tangential (rather than radial) to the cortical surface that we still have not used (Iivanainen et al., 2017).

We made use of the scanner‐cast here in order to provide some ground‐truth on sensor position and orientation. However, some skew in the position of the cast on the head is possible (we estimated this to be around ±3 mm, ±5°). We do not know therefore whether to attribute the final discrepancy (4 mm) between scanner‐cast measurements and algorithm estimates position to the cast or the algorithm. However, we note that the algorithm gives us posterior confidence bounds on the array location of better the 0.1019 cm^3^. We see one use of this algorithm is to further refine our geometrical estimates from the scanner‐cast.

## Data Availability

The data that support the findings of this study are available from the corresponding author upon request.

## METROPOLIS SEARCH

### 1

Based on the Metropolis algorithm, we adapted the approach presented in (López et al., 2012) to estimate the true location of the sensor array with respect to the brain:

1. Select a random sample from the prior over possible sensor geometries *h*_0_~*p*(*h*) and solve the EBB reconstruction for that geometry. This returns a Free energy value *F*(*h*_0_).
2. Use a Gaussian distribution to obtain the new array position near to the position computed on the previous step *h*_*k* − 1_ : *h*^′^~*N*(*h*^′^; *h*_*k* − 1_, *σ*^2^*I*).
3. Perform EBB reconstruction on the new location of the array and calculate the ratio *r* with the new Free energy values:

$$r=\frac{p\left(Y|h^{\prime }\right)p\left(h^{\prime }\right)}{p\left(Y|h_{k-1}\right)p(h_{k-1)}}=\exp\left(F\left(h^{\prime }\right)-F\left(h_{k-1}\right)\right)\frac{p\left(h^{\prime }\right)}{p\left(h_{k-1}\right)}$$

The ratio is given by the comparison of *log* evidence between the previous reconstruction *p*(*Y*| *h*_*k* − 1_), and the proposed one *p*(*Y*| *h*^′^), where each is also weighted by the prior. A ratio larger than one means that the proposed geometry *h*^′^ has more model evidence than the previous one.

4. Take a decision: if *r* > 1 (the new step has higher Free energy), then the new value is higher and accepted *h*_*k*_ = *h*^′^ ; if *r* < 1, then the new value is compared with a random sample obtained from the uniform distribution: *β*~*U*(0, 1). If *β* < *r* the parameters are accepted, or rejected otherwise: *h*_*k*_ = *h*_*k* − 1_. Allowing such transitions enables the algorithm to escape from local maxima.
5. Return to the second step and repeat until convergence. After an initial burn‐in period (first half of data samples), the samples together comprise an approximate posterior distribution over the array locations.

## BAYESIAN MODEL AVERAGING

### 1

The following BMA algorithm is used to provide an estimate of the posterior mean $\hat{J}$. It was set‐up for *k* = 10,000 iterations.

- For the current iteration *k*, pick a random array geometry from the posterior distribution *h*_*k*_~*p*(*h*| *Y*)
- For the selected array geometry *h*_*k*_ (i.e., its corresponding gain matrix), compute the estimated values of the neural activity *J*_*t*_, and its posterior covariance ∑_*k*_ with Equation (2).
- Obtain a normal random variable with mean ${\hat{J}}_{k}$ and covariance ${\left(\sum_{J}\right)}_{k}:{\hat{J}}_{t}\sim N\left({\hat{J}}_{t}|{\hat{J}}_{k},\left({\left(\sum_{J}\right)}_{k}\right)\right)\;$and save.
- Update *k* and go back to step a. until *k* = 10,000.
- Obtain the mean of the random variables. $\hat{J}=\sum {\hat{j}}_{k}/k$
